# A retrospective case-control service evaluation of CAARMS scores of patients with autism in York EIP, compared to age matched controls

**DOI:** 10.1192/bjo.2021.203

**Published:** 2021-06-18

**Authors:** Daniel Whitney, Stephen Wright

**Affiliations:** Tees Esk and Wear Valleys NHS Foundation Trust

## Abstract

**Aims:**

Studies show the prevalence of Autism Spectrum Conditions in Early Intervention in Psychosis (EIP) populations is 3.6-3.7%, compared to approximately 1-1.5% in the general population. The CAARMS (Comprehensive Assessment of At Risk Mental States) is a national tool used by EIP services as a screening tool to bring patients into services and stratify their symptoms to determine what pathway may be most appropriate (First Episode Psychosis pathway (FEP) or At Risk Mental State pathway (ARMS)). As far as we are aware the CAARMS has not been validated in an autistic population. It is our view that several of the questions in the CAARMS may be interpreted differently by people with autism, thus affecting the scores. The aim of this evaluation was to identify whether CAARMS scores differ between patients diagnosed with autism and matched controls in York EIP.

**Method:**

From their mental health records, we identified all patients in the service with a diagnosis of autism. We then compared the CAARMS scores, at the time of referral, to those of age matched controls (matched by being in the age range 16-30) without an autism diagnosis, using continuous sampling by date of referral.

**Result:**

14 patients in the service had a diagnosis of autism and had completed a CAARMS. CAARMS domains are all scored between 0 and 6 (indicating increasing severity or frequency). Compared to the age matched controls, autistic patients had a higher mean difference in their scores for ‘Non-Bizarre Ideas’ (mean difference of 0.86 for severity and 0.57 for frequency) and ‘Disorganised Speech’ (mean difference of 0.28 for severity and 0.57 for frequency). These results did not reach statistical significance which was unsurprising given the sample size. The gender split between groups was similar.

**Conclusion:**

Our evaluation suggests a difference in CAARMS scores between patients in our service with a diagnosis of autism and those without. A larger study would be needed to confirm a statistically significant difference and multicentre results would be needed as evidence of generalisability. However, if such a difference were confirmed it might question the validity of CAARMS in autistic patients or suggest that modifications, perhaps in the form of reasonable adjustments to the questions or scoring, were needed to increase the validity in this population. We would suggest that spending extra time checking the patient has understood the intended meaning of the questions in the CAARMS may increase validity, particularly in the ‘Non-Bizarre Ideas’ domain.

